# Radiation in an emergency situation: attempting to respect the patient’s beliefs as reported by a minor

**DOI:** 10.1186/s12910-023-00962-5

**Published:** 2023-10-04

**Authors:** Tetsuya Yumoto, Takashi Hongo, Yasuhiro Koide, Takafumi Obara, Kohei Tsukahara, Hiromichi Naito, Atsunori Nakao

**Affiliations:** Department of Emergency, Critical Care, and Disaster Medicine, Faculty of Medicine, Dentistry, and Pharmaceutical Sciences, Okayama University, 2-5-1 Shikata-cho, Kita- ku, Okayama, 700-8558 Japan

**Keywords:** Emergency service, Informed consent, Radiation, Treatment refusal

## Abstract

**Background:**

Each individual’s unique health-related beliefs can greatly impact the patient-clinician relationship. When there is a conflict between the patient’s preferences and recommended medical care, it can create a serious ethical dilemma, especially in an emergency setting, and dramatically alter this important relationship.

**Case presentation:**

A 56-year-old man, who remained comatose after out-of-hospital cardiac arrest, was rushed to our hospital. The patient was scheduled for emergency coronary angiography when his adolescent daughter reported that she and her father held sincere beliefs against radiation exposure. We were concerned that she did not fully understand the potential consequences if her father did not receive the recommended treatment. A physician provided her with in depth information regarding the risks and benefits of the treatment. While we did not want to disregard her statement, we opted to save the patient’s life due to concerns about the validity of her report.

**Conclusions:**

Variations in beliefs regarding medical care force clinicians to incorporate patient beliefs into medical practice. However, an emergency may require a completely different approach. When faced with a patient in a life-threatening condition and unconscious, we should take action to prioritize saving their life, unless we are highly certain about the validity of their advance directives.

**Supplementary Information:**

The online version contains supplementary material available at 10.1186/s12910-023-00962-5.

## Background

Physicians occasionally confront a substantial dilemma when a patient refuses recommended medical treatment based on their religious or cultural beliefs. This dilemma becomes more pronounced and complex in emergency circumstances or when a patient is unconscious, and treatment is deemed essential [1]. In general, in current medical practice, adult patients who are assumed to be capable of decision-making have the right to refuse medical treatment [2]. Inevitably, an extreme conflict can arise among health care professionals when there is uncertainty regarding a patient’s advance directives or wishes and significant urgency for a treatment that a patient might oppose. Legal ramifications reinforce this concern. In such challenging scenarios, it is crucial for clinicians to thoroughly evaluate evidence when patients decline capacity assessments. This ensures the patient’s refusal is both informed and autonomous, balancing respect for autonomy with well-being [3].

Here, we describe how we handled decision-making when an unconscious patient presented to the emergency department with a philosophical belief against radiation exposure that was unexpectedly reported by his adolescent daughter.

## Case presentation

A 56-year-old man was brought to our tertiary medical center unconscious after collapse at home from out-of-hospital cardiac arrest and successful resuscitation. Return of spontaneous circulation was achieved after the delivery of 2 shocks with an automated external defibrillator. On arrival at the emergency department, the patient was in deep coma with Glasgow Coma Scale score of 3. An electrocardiogram revealed ST-segment elevation in lead augmented vector right, depression in leads V3-V6, and hypokinesis of the anterior cardiac wall. An acute myocardial infraction was suspected. Prompt treatment was essential, otherwise it would be fatal. The patient was intubated and immediately scheduled for a coronary arteriography (CAG) and revascularization, which involve certain amount of radiation exposure.

The patient presented with his 14-year-old daughter and 11-year-old twin sons and without any adult relatives or legally authorized representatives. His wife had died from gastric cancer 3 years earlier. The patient had undergone a left lobe thyroidectomy for a pT1N0M0 papillary thyroid carcinoma 8 years prior. Quite unexpectedly, when providing collateral history, his daughter revealed that she and the patient held sincere beliefs against any radiation exposure. She refused to approve with any medical procedures requiring radiation exposure for her father. Her testimony was deemed credible because she stated that he and his family evacuated far from his hometown following the Fukushima nuclear disaster. This was despite his residential area being publicly declared scientifically safe and not warranting evacuation. She further mentioned that he had previously refused any procedures involving radiation. The daughter also reported that, as far as she knew, the patient did not have a formal document to expressing this belief. We were faced with an apparent conflict between the daughter’s testimony regarding the patient’s strong opposition to radiation exposure and the principle of beneficence: respecting a patient’s potential opposition to radiation exposure or proceeding with the best medical care including emergency CAG.

While expressing empathy for the 14-year-old’s concerns, an attending physician patiently and clearly explained the circumstances. The attending physician discussed with the daughter that her father’s condition was life-threatening and that the radiological examination and intervention were crucial to save her father’s life. This resonated with the daughter. She eventually agreed to permit definitive treatment to her father for the suspected acute myocardial infarction, keeping radiation doses “as low as possible”. We attempted to contact the patient’s older sister repeatedly, but she did not answer the calls. Collectively, the care team reached a consensus that we should proceed with our planned treatment as we considered this the best possible medical care.

Emergency CAG showed subtotal occlusion of left main coronary artery. The patient was diagnosed with acute myocardial infarction and percutaneous coronary intervention for the left main coronary artery disease was performed. Standard protocols, designed to minimize radiation exposure as a matter of routine, were followed. Target temperature management was applied in the intensive care unit (ICU) for 24 h. Three days after admission, the patient was able to follow commands and was successfully extubated. Screening tests revealed that neurocognitive impairment was minimal.

The day after ICU admission, the medical team was finally able to reach the patient’s sister and explained the overall situation. She described what he had been like in detail; he has always been extremely particular about foods; he has avoided foods that are potentially radiation-contaminated as much as possible since a young age. She did not have a belief against radiation exposure, and completely agreed with all the actions we had taken. Because the patient was comatose and no other legally authorized representatives were available besides her, we acknowledged that she was a surrogate. Although she did not actively participate in decision-making, she would have been an appropriate and legally designated surrogate if we had been able to reach her earlier. We still felt comfortable with her agreeing with our decisions. Once the patient regained consciousness, he accepted and appreciated the treatment he had received to save his life. He disclosed that he had previously declined screening procedures requiring radiation and declined further procedures requiring radiation exposure. Our ethics committee reviewed the case and determined that the team’s decision and decision-make process were reasonable. The patient was discharged from the ICU after 10 days and returned home 4 days later without any complications.

A telephone interview was conducted with the patient 2 months after the event. He was still grateful for the actions we had taken. He mentioned that his belief against radiation exposure was not derived from a religious belief but was still a fervent and profound belief. The patient stated that he would permit the best possible medical care with a “minimum” radiation exposure level if a similar situation were to arise in the future.

## Discussion

This case illustrates an important clinical scenario requiring urgent medical decision-making in a situation with a considerable ethical dilemma. Although the patient’s adolescent daughter voicing that he had a sincere belief against radiation exposure produced significant confusion, the uncertainty regarding this information and the immediate threat to the patient’s life guided the care team to provide life-saving treatment despite the requirement for radiation exposure. Later, we identified a surrogate decisionmaker, to whom we justified the treatment. We fully informed the patient after his recovery and delivered consistent support to his family with review by the ethics committee.

Each patient has their own beliefs, values, and perspectives that clinicians must respect and take into consideration to maintain the patient-clinician relationship, as the quality of patient-clinician communication has an enormous impact on subsequent care, patient satisfaction, and healthcare outcomes [4]. Notably, it is important to recognize the diverse origins of beliefs that patients may hold. These can range from medically informed perspectives to deeply-held philosophical or personal beliefs. While these categories can sometimes intersect, balancing them is crucial in healthcare settings to ensure both respect for the patient and optimal medical care. However, this communication framework cannot be utilized when the patient is incapacitated, unconscious, and in some time-sensitive, life-threatening situations. A situation can become problematic when a patient’s beliefs are extreme enough to markedly affect health care and are substantially discrepant from the recommended medical treatment. A well-known example is the legal and ethical dilemma for emergency healthcare professionals when a Jehovah’s Witness presents with ongoing or potentially severe bleeding. Blood transfusions should not be avoided in life-threating conditions if clinicians perceive that the validity of a blood-refusal card or advance directives is uncertain [5]. Meanwhile, it can be argued that verbally expressed wishes of an individual should be consistently honored because they may represent the patient’s genuine preferences for medical care. Legally, it might be possible to ignore such declarations, but doing so could raise significant concerns from an ethical standpoint (6). Hurst’s argument indicates that when a patient refuses to cooperate in a capacity assessment, clinicians should carefully evaluate all available evidence to determine that the patient is competent. In critical situations with high stakes, they should seek a higher degree of certainty to be confident that the patient’s refusal is genuinely autonomous and well-informed. This approach is essential for achieving a balance between honoring the patient’s autonomy and ensuring their well-being in cases where their capacity to make decisions is uncertain or in doubt [3].

In the case presented here, the patient faced immediate danger and the potential sacrifices he was willing to make to avoid radiation exposure were unclear; a legal document to express this patient’s belief did not exist. Furthermore, situations when there is no legal surrogate available can be challenging for clinicians as well. Doubts emerged regarding the validity of the unexpected statement from the patient’s adolescent daughter about his and his family’s refusal of radiation exposure. From both medical and ethical standpoints, we believe our decision to prioritize his life was justified. Concurrently, considering the daughter was perceived to be capable, mature, and trustworthy, we made every effort to ensure she was not overlooked or left out of the decision-making process. Although she was not legally liable, the information she provided could not be disregarded. When attempting to explain about the procedure, however, we did not think that she fully appreciated the devastating consequences if the patient did not promptly receive the recommended treatment. Indeed, after thorough, patient, and respectful back-and-forth communication, she appreciated the recommended therapies to preserve her father’s life over continued refusal of any radiation exposure. Even if she had initially refused radiation exposure, we would have proceeded with the treatment to save her father’s life and then explained the rationale or reasoning behind our decision.

Emergency patients are more likely to seek accurate diagnoses rather than attempt to obtain information about radiation exposure [7, 8]. Nonetheless, clinicians should be mindful that some individuals have fears surrounding or beliefs against radiation exposure, which the physician will likely consider unwarranted. Effective communication is needed to elicit the reasons why a patient is refusing medical radiation exposure, and to explain and discuss benefits, potential harms, and alternatives.

In the emergency department, waiving informed consent for medical care is permissible, but only in situation where the patient is unconscious, there is no surrogate available, and the patient faces imminent risk of death without a life-saving procedure [9]. Otherwise, there is an ethical duty to adhere to informed consent procedures as closely as possible. Patients, their family, or their representatives may reveal beliefs that may be regarded as a prejudiced or incomprehensible to the treating clinicians. Even though a significant ethical conflict may arise when there is a profound gap between the patient’s and the clinician’s views with limited time to initiate treatment, the healthcare team should make every effort to provide safe care and build a successful patient/family-clinician relationship. Physicians need to provide clear information and a professional perspective on recommended medical care and its benefits and risks.

## Conclusions

Clinicians may encounter situations where an emergency medical care is interrupted by a sudden report from the patient’s family that they have a sincere belief against a specific medical procedure, which can create major confusion or an ethical dilemma. Specifically, when dealing with unconscious patients in life-threatening conditions, we should opt to preserve the patient’s life unless there is a high degree of certainty regarding the validity of advance directives. More importantly, respectful discussion and thorough explanations can be crucial to maintaining the therapeutic relationship.

### Electronic supplementary material

Below is the link to the electronic supplementary material.

Supplementary Material 1

## Data Availability

Not applicable.
