# Supplementary material for: Radiation in an emergency situation: attempting to respect the patient’s beliefs as reported by a minor
Source: BMC Med Ethics. 2023 Oct 4;24:80. doi: 10.1186/s12910-023-00962-5 (PMC10552384; doi:10.1186/s12910-023-00962-5)
Supplement: Supplementary file 1 — Supplementary Material 1 [file 12910_2023_962_MOESM1_ESM.pdf]

CARE Checklist of information to include when writing a case report

CARE case report guidelines

(cc) BY-NC-ND

Topic	Item	Checklist item description	Reported on Line
Title	1	The diagnosis or intervention of primary focus followed by the words "case report"	P. 1, line 2 to 3
Key Words	2	2 to 5 key words that identify diagnoses or interventions in this case report, including "case report"	P. 2, line 22
Abstract (no references)	3a	Introduction: What is unique about this case and what does it add to the scientific literature?	P. 2, line 2 to 6
	3b	Main symptoms and/or important clinical findings	P. 2, line 6 to 10
	3c	The main diagnoses, therapeutic interventions, and outcomes	P. 2, line 13 to 15
	3d	Conclusion—What is the main "take-away" lesson(s) from this case?	P. 2, line 16 to 20
Introduction	4	One or two paragraphs summarizing why this case is unique (**may include references**)	P. 3, line 1 to 16
Patient Information	5a	De-identified patient specific information.	P. 3, line 19 to 21
	5b	Primary concerns and symptoms of the patient.	P. 3, line 21 to 23
	5c	Medical, family, and psycho-social history including relevant genetic information	P. 4, line 7 to 8
	5d	Relevant past interventions with outcomes	P. 4, line 7 to 8
Clinical Findings	6	Describe significant physical examination (PE) and important clinical findings.	P. 3, line 22 to 23
Timeline	7	Historical and current information from this episode of care organized as a timeline	P. 3, line 19 to 22
Diagnostic Assessment	8a	Diagnostic testing (such as PE, laboratory testing, imaging, surveys).	P. 3 line 23 to 25
	8b	Diagnostic challenges (such as access to testing, financial, or cultural)	P. 4 line 5 to 21
	8c	Diagnosis (including other diagnoses considered)	N/A
	8d	Prognosis (such as staging in oncology) where applicable	N/A
Therapeutic Intervention	9a	Types of therapeutic intervention (such as pharmacologic, surgical, preventive, self-care)	P. 5, line 7 to 9
	9b	Administration of therapeutic intervention (such as dosage, strength, duration)	N/A
	9c	Changes in therapeutic intervention (with rationale)	N/A
Follow-up and Outcomes	10a	Clinician and patient-assessed outcomes (if available)	P. 5, line 25 to P. 6 line 1
	10b	Important follow-up diagnostic and other test results	P. 6, line 7 to 12
	10c	Intervention adherence and tolerability (How was this assessed?)	P. 6 line 7 to 12
	10d	Adverse and unanticipated events	N/A
Discussion	11a	A scientific discussion of the strengths AND limitations associated with this case report	P. 6, line 14 to P. 9 line 14
	11b	Discussion of the relevant medical literature **with references**.	P. 6, line 14 to P. 9 line 14
	11c	The scientific rationale for any conclusions (including assessment of possible causes)	P. 9 line 17 to 24
	11d	The primary "take-away" lessons of this case report (without references) in a one paragraph conclusion	P. 9 line 17 to 24
Patient Perspective	12	The patient should share their perspective in one to two paragraphs on the treatment(s) they received	P. 6 line 7 to 12
Informed Consent	13	Did the patient give informed consent? Please provide if requested	Yes ☑ No ☐
